# Efficacy of *Perilla frutescens* (L.) Britton var. *frutescens* extract on mild knee joint pain: A randomized controlled trial

**DOI:** 10.3389/fphar.2023.1114410

**Published:** 2023-03-14

**Authors:** NamHoon Kim, Si-Yeon Kim, Sang-Woo Kim, Jung Min Lee, Sung-Kyu Kim, Mi-Houn Park, Ki-Hwan Kim, Minseok Oh, Chang-Gue Son, In Chul Jung, Eun-Jung Lee

**Affiliations:** ^1^ Department of Korean Rehabilitation Medicine, College of Korean Medicine, Daejeon University, Daejeon, Republic of Korea; ^2^ Clinical Trial Center, Daejeon Korean Medicine Hospital of Daejeon University, Daejeon, Republic of Korea; ^3^ SFC Bio Co., Ltd, Cheonan-si, Republic of Korea; ^4^ Institute of Bioscience and Integrative Medicine, Department of Korean Medicine, Daejeon University, Daejeon, Republic of Korea; ^5^ Department of Oriental Neuropsychiatry, College of Korean Medicine, Daejeon University, Daejeon, Republic of Korea

**Keywords:** knee joint pain, *Perilla frutescens* (L.) Britton var. *frutescens*, randomized-controlled trials, visual analogue scale, western Ontario and McMaster universities osteoarthritis

## Abstract

**Objectives:** This study aimed to evaluate the clinical efficacy and safety of PE extracts developed for the purpose of relieving pain and improving knee joint function on semi-healthy people with mild knee joint pain.

**Methods:** A randomized, double-blind, two-arm, single-center, placebo-controlled clinical trial was conducted. Individuals with knee joint pain and a visual analogue scale (VAS) score < 50 mm were included in the study, and participants with radiological arthritis were excluded. Participants were administered either PFE or a placebo capsule (700 mg, twice a day) orally for eight weeks. The comparisons of the changed VAS score and Western Ontario and McMaster Universities Osteoarthritis (WOMAC) scores between the PFE and placebo groups were primary outcomes, while the five inflammation-related laboratory tests including cartilage oligomeric matrix protein, cyclooxygenase-2, neutrophil and lymphocyte ratio, high sensitive C-reactive protein, and erythrocyte sedimentation rate were secondary outcomes. Also, a safety assessment was done.

**Results:** Eighty participants (mean age, 38.4 ± 14.0, male: female, 28:52) were enrolled; 75 completed the trial (PFE 36 and placebo 39). After eight weeks, both VAS and WOMAC scores were reduced in the PFE and placebo groups. The changed scores were significantly higher in the PFE group compared to the placebo group: 19.6 ± 10.9 vs. 6.8 ± 10.5; VAS scores (*p* < 0.001), and 20.5 ± 14.7 vs. 9.3 ± 16.5; total WOMAC scores (*p* < 0.01) including the sub-scores for pain, stiffness, and functions. No significant changes were reported in the five inflammation-related laboratory parameters. All adverse events were considered minor and unlikely to result from the intervention.

**Conclusion:** Eight weeks of PFE intake was more effective than placebo in reducing knee joint pain and improving knee joint function in sub-healthy people with mild knee joint pain, and there were no major safety concerns.

**Clinical Trial Registration:** https://cris.nih.go.kr/cris/search/detailSearch.do?search_lang=E&focus=reset_12&search_page=M&pageSize=10&page=undefined&seq=23101&status=5&seq_group=19745, identifier CRIS: KCT0007219

## 1 Introduction

The knee is a modified hinge joint, having both tibiofemoral and patellofemoral components. The human knee joint evolved to adapt to bipedalism more than 300 million years ago (Dye, 2003). The continuous and repetitive stress from everyday activities, such as jogging, playing sports, working, standing, or sitting, makes the knee joints susceptible to problems such as injury or even osteoarthritis (Hartmann et al., 2013). Accordingly, the knee joint is the site at which the “wear-and-tear” type of arthritis occurs most commonly (Darlow et al., 2018), and knee-joint pain has a 22.9% global prevalence in individuals aged 40 and over (Cui et al., 2020).

On the other hand, knee pain is a typical symptom of mechanical disorders, likely injury, or osteoarthritis (Previtali et al., 2022). In addition, knee pain can commonly occur in conditions of functional overload prior to progression to those mechanical problems (Felson, 2013), where its prevalence rate is approximately 30% of adults worldwide (Peat, 2001; Nguyen et al., 2011). Knee pain prevalence generally increases with age, especially in females; 46.2% as a 1.8-fold female dominance in the Korean population aged ≥ 50 years (Kim et al., 2011).

Knee joint pain increases health risks such as fall-related injuries and limits physical movement and daily activities (Svensson et al., 2000; Lajoie and Gallagher, 2004; Lee et al., 2021). Chronic knee pain is often the result of several causes or conditions and needs certain treatments (Peat, 2001). Recommended treatments for knee pain include exercises, physiotherapy, or pharmacological medicines such as non-steroidal anti-inflammatory drugs (NSAIDs) or acetaminophen (AAP) (Conaghan et al., 2008). Several researchers have been interested in nutritional supplements and functional foods as an option to care for knee joint pain and disability (Ginnerup-Nielsen et al., 2015; Suzuki et al., 2016; Zdzieblik et al., 2017; Rao et al., 2019). Users expect that these multiple compounds provide benefits *via* targeting multiple pathways of knee dysfunction, including multifactorial cartilage degradation, as an alternative to pharmacological interventions that exert mainly a monomodal mode of action (Ameye and Chee, 2006).

*Perilla frutescens* (L.) Britton var. *Frutescens* (PF), also known as ‘Zisu’ in Chinese, ‘Cha-jo-ki’ or ‘So-yeop’ in Korean, and ‘Jiso’ in Japanese, is an important food ingredient and medicinal plant in East Asia (Kim et al., 2017). Its recognized bioactivities include antioxidant action Meng et al., 2008; Takahashi et al., 2011; Ahmed et al., 2022), immune control (Kwon et al., 2002), skin wound healing (Kim et al., 2021), anti-glomerulonephritis action (Makino et al., 2001), and anti-arthritis action (Jin et al., 2019). In particular, a marker compound named “Isoegomaketone” from PF has an excellent anti-inflammatory response (Kim et al., 2020), which is effective for treating arthritis and joint swelling (Jin et al., 2017). However, the aforementioned data comes from pre-clinical studies and the efficacy and safety of PF in humans has not been determined.

This trial aimed to evaluate PE extracts (PFE)’s clinical efficacy of alleviating pain and improving function of knee joint and its safety on semi-healthy people who have mild knee joint pain.

## 2 Materials and methods

### 2.1 Participants

A total of 80 participants were enrolled for this trial at Daejeon Korean medicine hospital of Daejeon University from July 2020 to January 2021. We included male and female patients aged 20–75 years who complained of knee pain with a VAS score ≤ 50 mm (without recent any type of joint injury or similar episode; 0 and 100 indicate ‘no pain’ and ‘unbearable pain’, respectively) but without a Kellgren-Lawrence (K-L) grade ≥ 3 on plain radiographs. The range of knee pain in this study was defined as medial, lateral or peripatella pain but no radiation pain. (www.merriam-webster.com, Nice Clinical Guidelines, 2022) We excluded any participant who had: 1) moderate to severe knee pain and taking arthritis medications; 2) moderate to severe arthritis as identified on plain radiographs; 3) a medical history of knee arthroplasty surgery; 4) been diagnosed with other musculoskeletal disorders other than knee joint pain and is being treated for pain; 5) any issues in taking products containing PFE, including allergic reaction; or 6) any other musculoskeletal pain other than knee pain; 7) plans of pregnancy or breastfeeding; and 8) heart, kidney, liver, and other organ disease. Participants were not permitted to take medication or consume health functional foods that may have an impact on the joint health. Detailed inclusion and exclusion criteria are available at CRIS: KCT0007219 (available at: https://cris.nih.go.kr/cris/search/detailSearch.do?search_lang=E&focus=reset_12&search_page=M&pageSize=10&page=undefined&seq=23101&status=5&seq_group=19745). Participants were provided with voluntary written informed consent.

In this study, the sample size was calculated based on a two-tailed alpha level of 0.05 and a power level of 0.80 respectively using G*power (version 3.1.9.2., Department of Psychology, Germany) (Faul et al., 2009). Also, the minimal detectable effect size was *r* = 0.7, and the target sample size was 80 assuming a 20% dropout rate.

### 2.2 Clinical trial design

This study was conducted as a randomized, double-blind, and placebo-controlled trial (RCT) at Daejeon Korean Medicine Hospital in South Korea. The purpose of this RCT was to evaluate the efficacy and safety of an eight-week administration regime of PFE on semi-healthy people with mild knee joint pain. This trial has been conducted in accordance with the Declaration of Helsinki, and the study protocol was approved by the Institutional Review Board (IRB) of Daejeon University (DJDSKH-20-BM-11).

The automatically generated random numbers (RNs) (from 1 to 80) were allocated to participants in the order of enrolling in the trial, and the intervention (PFE or placebo) corresponding to the RN was given under double blindness (participants, assessor, principal investigator, and any officer, including statistician). Participants took PFE capsules or placebos (twice a day) for eight weeks. PFE capsules (700 mg/capsule, Lot number: FSP 2020070) are composed of PFE (486 mg, 34.7%) and an excipient. The excipient portion was a combination of soybean oil (48.3%), palm oil (11.3%), beeswax (3.8%), and soybean lecithin (2.0%). Meanwhile, the placebo capsule (700 mg/capsule, Lot number: FSP 2020071) consisted of only excipients (soybean oil 83%, palm oil 11.25%, beeswax 3.75%, and soybean lecithin 2.00%). PFE and placebo capsules were manufactured and supplied by Suheung Co., Ltd. (Osong-eup, Cheongju-si, South Korea). There were no differences in color, odor, consistency, packaging, or labeling between the PFE and placebo foods.

### 2.3 Efficacy assessments

The primary efficacy assessment employed pain and osteoarthritis-related parameters; VAS for knee joint pain using a 100 mm drawing bar (0 and 100 indicate ‘no pain’ and ‘worst possible, unbearable pain’, respectively) and Western Ontario and the McMaster Universities Osteoarthritis (WOMAC) score evaluating pain, stiffness, and function of the knee joint (Salaffi et al., 2003). These parameters were measured at baseline, 4 weeks, and 8 weeks. The difference of both VAS and WOMAC scores measured between at baseline and at week 8 was determined as the primary outcome of this study.

As secondary outcomes, five inflammation and cartilage-related parameters were included: cyclooxygenase-2 (COX-2), high sensitive C-reactive protein (HS-CRP), erythrocyte sedimentation rate (ESR), neutrophil and lymphocyte ratio, and cartilage oligomeric matrix protein (COMP). These parameters were measured by blood tests in fasting participants at baseline and at week 8.

### 2.4 Safety assessments

For the safety assessment of PFE, red blood cells, white blood cells, hemoglobin, hematocrit, platelets, neutrophils, and lymphocytes were analyzed as hematological tests. In addition, biochemical tests such as serum total protein, albumin, alanine aminotransferase, aspartate aminotransferase, alkaline phosphatase (ALP), blood urea nitrogen (BUN), total bilirubin, creatinine, creatine kinase, glucose (fasting blood sugar; FBS), total cholesterol, high-density lipoprotein cholesterol (HDL-Cholesterol), triglyceride, uric acid, sodium (Na), potassium (K), and chlorine were analyzed, along with routine urine analyses (pH, WBC, glucose, nitrate, protein, ketone, urobilinogen, bilirubin, and specific gravity) at baseline and eight weeks.

In addition, the participants’ vital signs [blood pressure (BP), pulse rate (PR), and body temperature (BT)] were recorded at all visits during the study. Every participant was required to report any adverse events during the trial.

### 2.5 Statistical analysis

Analyses of the efficacy of PFE in the population were mainly presented as a per-protocol (PP) set and compared with the full analysis (FAS) set, which was analyzed further. The safety of PFE was analyzed by a safety set.

Categorical variables such as sex, drinking, and smoking experiences of baseline characteristics were compared by chi-square test, and the distribution of the K-L grade was compared by Fisher’s exact test. Continuous variables within a group were compared by a paired *t*-test, while those between groups were compared by an independent two-sample *t*-test. Since baseline characteristics of age and WOMAC-pain between groups were statistically different, continuous variables of primary efficacy assessment outcomes were compared by analyzed by analysis of covariance (ANCOVA) using age and value at baseline as covariate for further analysis. Examples of continuous variables in this trial were age, height, weight, and efficacy assessment indexes. Furthermore, to compare primary efficacy assessment by week 0, week 4, week 8 we used repeated measures analysis of variance (ANOVA). A *p*-value of <0.05 was considered statistically significant. Statistical analysis was performed using the statistical analysis SAS software (version 9.4, SAS Institute Inc., Cary, NC, United States).

## 3 Results

### 3.1 Characteristics of participants

**FIGURE 1:**
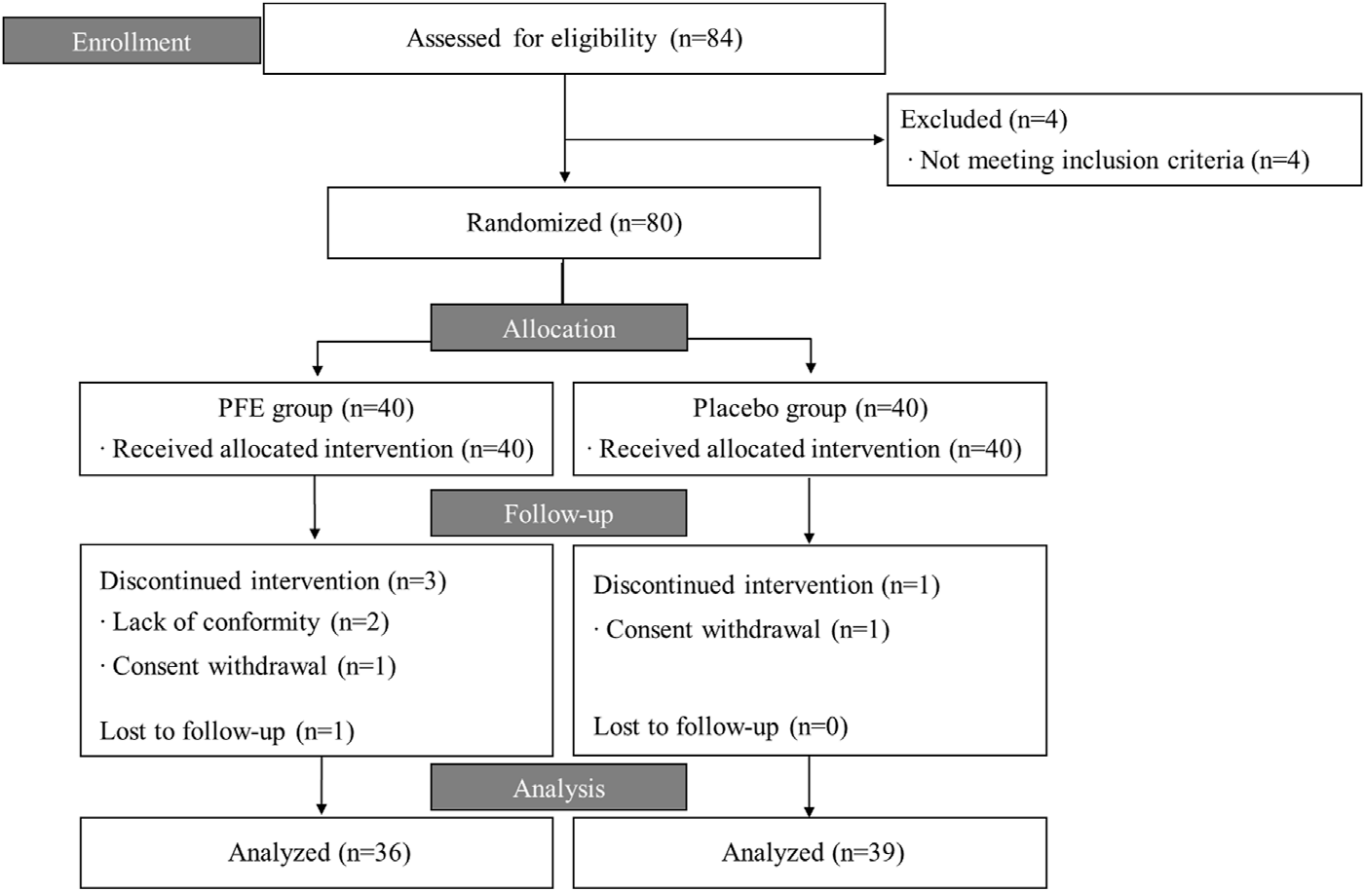
Flow chart of the trial process. PFE: *Perilla frutescens* (L.) Britton var. *frutescens*Extract.

This trial was conducted from July 2020 to January 2021. Of the 84 candidates screened, 80 participants fulfilling the inclusion criteria were randomized. 75 participants (36 in PFE, 39 in placebo) completed the trial, while five participants dropped out and were excluded from the PP analysis (Figure 1). Total 35% of participants were men and the mean age was 38.4 ± 14.0 years. There were no differences in the baseline characteristics among the groups, except for age. The mean age of the PFE group (42.3 ± 12.6) was significantly higher than the age of placebo group (34.5 ± 14.5) (Table 1).

**TABLE 1:** Baseline characteristics of participants.

Characteristics	PFE	Placebo	*p*-value
N. of subjects	40 *(36)*	40 *(39)*	> 0.05
N. of Male/Female (%)	14 (35) / 26 (65)	14 (35) / 26 (65)	> 0.05
	*12 (33) / 24 (67)*	*13 (33) / 26 (67)*	
Mean age (year)	42.3 ± 12.6 *(42.2 ± 12.6)*	34.5 ± 14.5 *(33.7 ± 13.9)*	< 0.05^*^
Mean BMI	23.4 ± 2.7 *(23.4 ± 2.8)*	22.4 ± 3.4 *(22.3 ± 3.4)*	> 0.05
N. of Kellgren-Lawrence grade 0/1/2 (%)	37 (93) / 2 (5) / 1 (1)	38 (95) / 2 (5) / 0 (0)	> 0.05
	*33 (92) / 2 (6) / 1 (3)*	*37 (95) / 2 (5) / 0 (0)*	
Alcohol use	22 (55) / 18 (45)	20 (50) / 20 (50)	> 0.05
Yes/No (n, %)	*20 (56) / 16 (44)*	*19 (49) / 20 (51)*	
Smoking	5 (13) / 35 (88)	5 (13) / 35 (88)	> 0.05
Yes/No (n, %)	*4 (11) / 32 (89)*	*5 (13) / 34 (87)*	

The characteristics of participants included in the PP analysis were written in *italics.* Data presented as mean ± standard deviation. Age and BMI are analyzed by an Independent two-sample *t*-test. Gender, alcohol use, and smoking are analyzed by the Chi-square test. Kellgren-Lawrence grade is analyzed by Fisher’s exact test. *Indicates the statistical significance *p* < 0.05 between PFE and placebo group. PFE: *Perilla frutescens* (L.) Britton var. *frutescens*Extract, N: number, BMI: body mass index.

### 3.2 Primary outcomes

After the eight-week administration regime, the VAS score in the PFE group (ΔVAS _PFE_ = 19.6 ± 10.9, *p* < 0.001) was significantly reduced by 12.8 ± 10.7 (*p* < 0.001) more than that in the placebo group (ΔVAS _placebo_ = 6.8 ± 10.5, *p* < 0.001).

Additionally, the WOMAC score was significantly mitigated by 11.2 ± 15.7 (*p* < 0.01) more in the PFE group (ΔWOMAC-total _PFE_ = 20.5 ± 14.7, *p* < 0.001) than the placebo group (ΔWOMAC-total _placebo_ = 9.3 ± 16.5, *p* < 0.01). The changes in scores of all WOMAC subscales (pain, stiffness, and function) were significantly greater in the PFE group than in the placebo (*p* < 0.01) group after eight weeks (Table 2).

**TABLE 2:** Changes in primary outcomes scores after 8-week intervention.

Variables		PFE (*n* = 36)	Placebo (*n* = 39)	Differences	*p*-value
VAS	Week 0	33.6 ± 8.6	31.9 ± 7.9	1.7 ± 8.3	
	Week 8	14.0 ± 10.7	25.1 ± 12.2	−11.1 ± 11.5	
	Change	19.6 ± 10.9***	6.8 ± 10.5***	12.8 ± 10.7	< 0.001***
WOMAC score					
Total	Week 0	31.4 ± 16.1	25.7 ± 15.7	5.7 ± 16.0	
	Week 8	10.8 ± 8.4	16.4 ± 13.1	−5.6 ± 11.1	
	Change	20.5 ± 14.7***	9.3 ± 16.5**	11.2 ± 15.7	0.005**
Pain	Week 0	6.8 ± 3.5	5.1 ± 3.0	1.7 ± 3.2^*^	
	Week 8	2.4 ± 1.9	3.1 ± 2.5	−0.7 ± 2.2	
	Change	4.4 ± 3.2***	2.0 ± 3.2***	2.4 ± 3.2	0.040*
Stiffness	Week 0	3.2 ± 2.0	2.8 ± 1.7	0.4 ± 1.8	
	Week 8	1.5 ± 1.7	1.9 ± 1.8	−0.4 ± 1.7	
	Change	1.9 ± 1.5***	0.9 ± 1.6**	1.0 ± 1.6	0.027*
Function	Week 0	21.2 ± 11.9	17.8 ± 12.1	3.4 ± 12.0	
	Week 8	6.9 ± 5.3	11.4 ± 9.6	−4.5 ± 7.8	
	Change	14.2 ± 11.2***	6.4 ± 12.2***	7.8 ± 11.7	0.003**

Data presented as mean ± standard deviation. Within-group comparisons of PFE and placebo groups are analyzed by paired *t*-test; week 0 vs. week 8. Differences are analyzed between groups are analyzed by analysis of covariance using age and value at baseline as covariate; PFE group vs. Placebo group. * *p* < 0.05, **: *p* < 0.01, ***: *p* < 0.001 indicates statistical significance between groups of PFE and placebo. PFE: *Perilla frutescens* (L.) Britton var. *frutescens*Extract, VAS: visual analogue scale, WOMAC: Western Ontario and McMaster Universities Osteoarthritis score.

**FIGURE 2:**
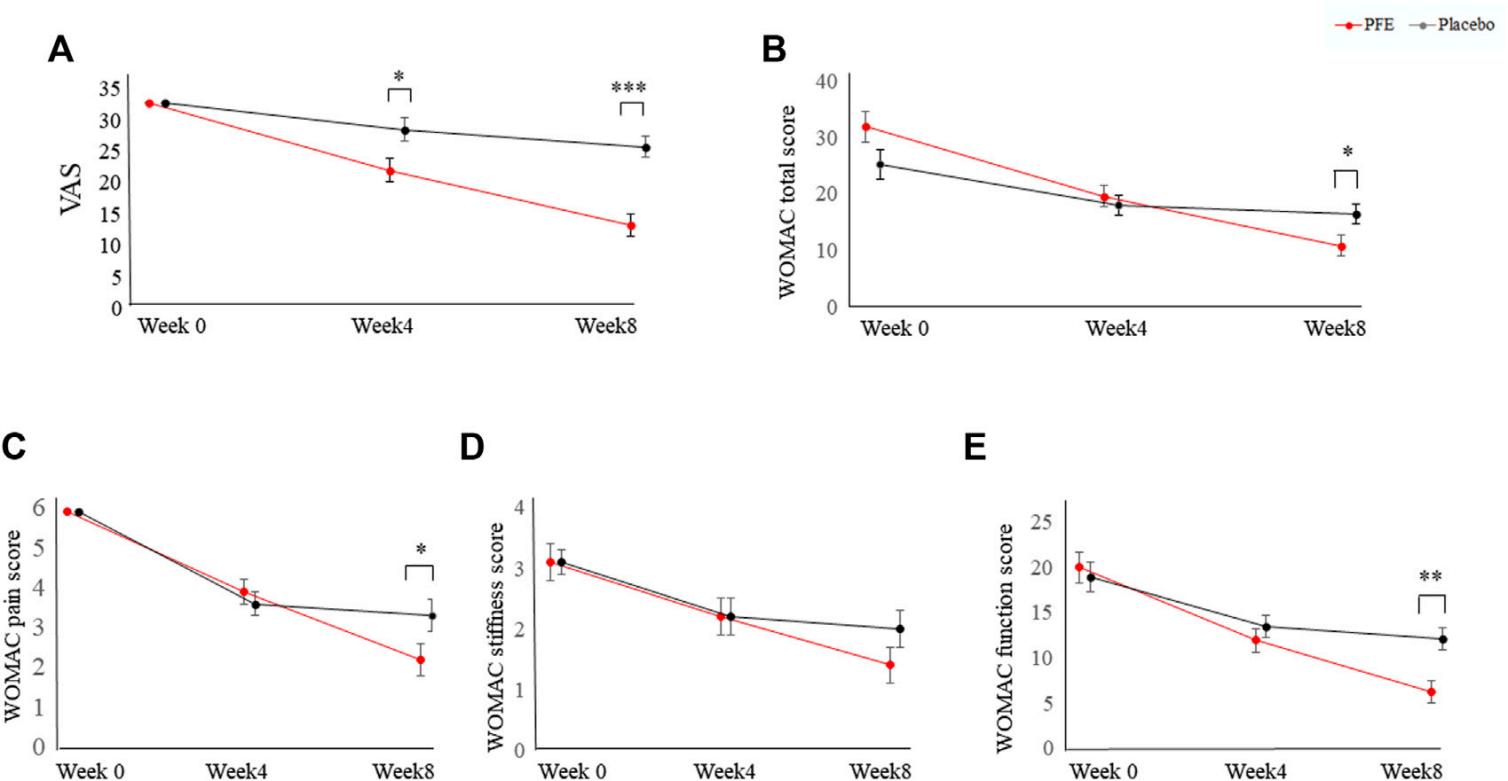
Changes in primary outcomes between the PFE group and the placebo group for eight weeks. **(A)** Changes in the VAS score; **(B)** Changes in the WOMAC total score; **(C)** Changes in the WOMAC pain score; **(D)** Changes in the WOMAC stiffness score; **(E)** Changes in the WOMAC function score. Data are corrected using age and value at baseline as covariance. *p*-values are analyzed by repeated measures analysis of variance (ANOVA). PFE: *Perilla frutescens* (L.) Britton var. *frutescens* Extract; VAS, visual analogue scale; WOMAC, Western Ontario and McMaster Universities Osteoarthritis *p < 0.05, **: *p* < 0.01, ***: *p* < 0.001 indicates statistical significance between the change values in the PFE group and the placebo group. *p*-values are calculated by repeated measures analysis of variance.

In the PFE group, the VAS and WOMAC scores continued to decline with a similar slope until week eight. However, the change in primary outcome value in the placebo group showed a tendency to stagnate after four weeks (Figure 2). There was no statistical difference between the PP set and the FAS set (data not shown).

### 3.3 Secondary outcomes

In the secondary efficacy outcomes, there were no statistically significant differences in the COMP, COX-2, HS-CRP and ESR levels, and the neutrophil and lymphocyte ratio in the PFE group compared with the placebo group at the end of the intervention (Table 3). Among them, the change of CRP levels in the PFE group after eight weeks had no statistical significance but showed a tendency to increase outside the normal range, and the change of ESR levels within the PFE group increased significantly (not *italics* in Table 3).

**TABLE 3:** Changes in secondary outcomes scores after 8-week intervention.

Variables		PFE (*n* = 36)	Placebo (*n* = 39)	Differences	*p*-value
		*PFE (n = 35)*#	*Placebo (n = 38)*#		
COMP (ng/mL)	Week 0	128.8 ± 42.9 *(129.3 ± 43.4)*	127.3 ± 45.9 *(127.1 ± 46.5)*	1.5 ± 44.5 *(1.5 ± 44.5)*	0.297 *(0.837)*
	Week 8	142.6 ± 56.4 *(143.8 ± 56.7)*	127.3 ± 43.7 *(126.6 ± 44.1)*	15.3 ± 50.2 *(17.2 ± 50.5)*	0.193 *(0.149)*
	Change	−13.7 ± 44.6 *(−14.6 ± 44.9)*	0.0 ± 33.1 *(0.5 ± 33.5)*	13.7 ± 39.0 *(13.7 ± 39.0)*	0.132 *(0.107)*
COX-2	Week 0	10.9 ± 14.3 *(11.1 ± 14.5)*	16.5 ± 41.0 *(16.8 ± 41.5)*	−5.6 ± 31.2 *(−5.6 ± 31.2)*	0.884 *(0.425)*
	Week 8	11.7 ± 14.2 *(11.8 ± 14.3)*	13.5 ± 23.2 *(13.7 ± 23.5)*	−1.8 ± 19.4 *(−1.9 ± 19.6)*	0.676 *(0.678)*
	Change	−0.7 ± 4.5 *(−0.8 ± 4.6)*	3.0 ± 18.8 *(3.1 ± 19.1)*	3.7 ± 13.9 *(3.7 ± 13.9)*	0.232 *(0.232)*
Neutrophil	Week 0	2.5 ± 1.4 *(2.5 ± 1.4)*	2.3 ± 0.9 *(2.2 ± 0.9)*	0.2 ± 1.1 *(0.2 ± 1.1)*	0.339 *(0.333)*
/Lymphocyte ratio	Week 8	2.3 ± 1.0 *(2.2 ± 0.9)*	2.3 ± 1.0 *(2.3 ± 1.0)*	0.0 ± 1.0 *(−0.1 ± 1.0)*	0.906 *(0.659)*
	Change	0.3 ± 4.8 *(0.3 ± 1.4)*	0.2 ± 6.5 *(−0.1 ± 1.1)*	−0.1 ± 5.3 *(−0.1 ± 5.3)*	0.960 *(0.215)*
HS-CRP	Week 0	0.8 ± 1.0 *(0.8 ± 1.0)*	0.7 ± 0.7 *(0.6 ± 0.7)*	0.1 ± 0.9 *(0.1 ± 0.9)*	0.431 *(0.410)*
	Week 8	2.2 ± 8.8 *(0.7 ± 0.8)*	0.9 ± 1.5 *(0.8 ± 1.4)*	1.3 ± 6.2 *(−0.1 ± 1.2)*	0.390 *(0.718)*
	Change	−1.4 ± 8.7 *(0.1 ± 0.9)*	−0.2 ± 1.1 *(-0.2 ± 1.1)*	1.2 ± 6.0 *(1.2 ± 6.0)*	0.434 *(0.254)*
ESR	Week 0	12.5 ± 6.4 *(12.7 ± 6.4)*	12.7 ± 10.9 *(11.6 ± 8.5)*	−0.2 ± 9.0 *(-0.2 ± 9.0)*	0.917 *(0.545)*
	Week 8	14.3 ± 7.3 *(14.1 ± 7.2)*	13.0 ± 11.0 *(11.9 ± 9.0)*	1.3 ± 9.4 *(2.1 ± 8.2)*	0.537 *(0.270)*
	Change	−1.8 ± 5.2* *(−1.4 ± 4.6)*	−0.3 ± 3.6 *(−0.3 ± 3.6)*	1.5 ± 4.4 *(1.5 ± 4.4)*	0.143 *(0.278)*

Data presented as mean ± standard deviation. Within-group comparisons of PFE and placebo groups are analyzed by a paired *t*-test; week 0 vs. week 8. Differences are analyzed between groups by an independent two-sample *t*-test; PFE group vs. Placebo group. * Indicates the statistical significance *p* < 0.05 between within PFE group. # Indicates the statistical values reanalyzed except for 2 patients who have extraordinary change of inflammatory markers and *italics* in the table. PFE: *Perilla frutescens* (L.) Britton var. *frutescens*Extract, VAS: visual analogue scale, WOMAC: Western Ontario and McMaster Universities Osteoarthritis score.

After precisely analyzing the CRP and ESR levels in 75 participants, we confirmed that the random number (RN) 48 had an abnormally elevated HS-CRP value after eight weeks due to severe shin inflammation and RN 46 had an abnormally elevated ESR value due to chronic lung disease (Supplementary Figure S1). Excluding these two participants (RN 46 and 48), on analysing again, it has been found that HS-CRP levels in the PFE group showed a tendency to decrease in the normal range after eight weeks, and the change in ESR levels in the PFE group had no statistical significance (*italics* in Table 3). There were no statistically significant changes in the primary outcome (data not shown).

### 3.4 Safety assessments

Routine laboratory tests, urine analyses, and vital sign results did not show any significant changes after intervention and were within the normal range throughout the trial (data not shown).

Of the 80 participants, 14 participants experienced 15 episodes of adverse events during the intervention period. Six participants (seven cases) in the PFE group and eight participants (eight cases) in the placebo group were reported to have experienced adverse events. The difference in the number of participants who experienced adverse events was not statistically significant between the groups (*p* > 0.5, analyzed by chi-square test). All adverse events were considered minor and were not likely caused by the intervention (Supplementary Table S1).

## 4 Discussion

In an eight-week RCT format, we evaluated the PFE effects on knee joint pain in participants with mild knee discomfort. The average VAS and WOMAC scores in 80 participants were 31.1 ± 8.6 and 28.1 ± 17.2, respectively, indicating a severity of approximately 30% of the total score in both evaluation indicators. As two primary outcomes in the present RCT, both VAS and WOMAC scores were well correlated with each other (Pearson correlation coefficient 0.63, *p* < 0.001, data not shown), as observed in other studies, including those on moderate to severe knee osteoarthritis (KOA) (Wang, 2017; Guo et al., 2018).

While the VAS is an intuitive and persistent indicator of general pain intensity, the WOMAC score reflects pain and function during activity associated with KOA in three domains: pain (20 points), joint stiffness (8 points), and function (68 points) (Chiarotto et al., 2019). The average WOMAC function score of the participants in our study (19.4 ± 12.0) was worse than the average value of those aged 75 to 79 (14.3 ± 15.4) in the general population of 5,509 Australians (Bellamy et al., 2009). When we evaluated the 75 participants who completed the entire trial for the VAS score, the WOMAC stiffness score, physical examination, and K-L grade according to the American College of Rheumatology (ACR) clinical criteria (defined by pain plus 3 or more of 6 factors) (Altman et al., 1986), 68 participants met the criteria for mild level of KOA. Based on the above two findings, we could assume that the participants showed mild symptoms of KOA at an early stage, even though the average age of participants (38.4 ± 14.0 years) was lower than the generally known age of osteoarthritis morbidity (age > 45 years) (www.nice.org.uk, Knee, 2014).

Except for four participants in the PFE group (three had withdrawn consent and one had an adverse event) and one participant in the placebo group (withdrawn consent), 75 participants completed the entire trial course. The eight-week administration of PFE significantly reduced the VAS score of knee pain by 58.3% in the PFE group (ΔVAS = 19.6 ± 10.9), which was significantly greater than the change in the placebo group (21.3% reduction, *p* < 0.001) (Table 2; Figure 2). This effect was repeated as a similar pattern in the WOMAC scores (65.3% in PFE vs. 36.2% in placebo, *p* < 0.01, Table 2; Figure 2). The placebo effect in this study might be related to the mild pain symptoms experienced by the participants. Previous research suggests that the higher the placebo effect, the less severe the symptoms and the shorter the duration of the disease (Kirsch, 2013; Enck and Klosterhalfen, 2019). Adverse events reported by one withdrawn female participant in the PFE group were discomfort, such as swelling and tingling of the tongue. She had been suffering from a Korean somatization disorder (called Hwa-Byung), and the symptoms disappeared after acupuncture treatments. All adverse events, including a digestive system problem, were reported at similar levels in both groups (seven cases in PFE vs. eight cases in placebo), which indicate no relationship with the intervention (Supplementary Table S2).

Knee pain can be caused by a variety of functional or organic problems with structural components such as subchondral bone, meniscus, ligaments, tendons, and muscles (Netter and Dalley, 2003; Lane et al., 2011). One of the main causes of knee pain is KOA (Zeni et al., 2010), which is the consequence of repeated stress on the knee by wear and tear sequences, resulting in progressive loss of articular cartilage (Darlow et al., 2018). High body mass index (BMI) scores, women, and smoking act as risk factors for the onset and exacerbation of KOA (Blagojevic et al., 2010; Driban et al., 2015). The number of female participants in our study was 1.9 times that of male participants (female: male = 52: 28), while participants older than 50, with a BMI of > 25, and smoking had a higher initial pain score without any statistical significance (data not shown). When we analyzed the risk factor-related effects of PFE administration, VAS and WOMAC scores of women revealed greater changes than those of men, even without any statistical significance except for WOMAC pain (Supplementary Table S2). Participants with overweight (BMI > 23), based on the Asia-Pacific obesity criteria, (World Health Organization. Regional Office For The Western Pacific, International Association For The Study Of Obesity and International Obesity Task Force World Health orgnization, 2002) showed lesser changes in the primary outcome than participants with normal BMI, especially in the total and 3-domain score of WOMAC (*p* < 0.05). Age did not affect the changes in the VAS and WOMAC scores (Supplementary Table S2).

The traditional diagnostic method for KOA was to classify the presence of osteophyte and narrowed joint space into four K-L grades in radiological examination (Jang et al., 2021). It is, however, reported that knee joint pain appears before radiographic evidence of KOA in 50% of patients (Morozzi et al., 2007) and that no or doubtful stage of KOA can be abruptly progressed to a severe stage with osteophytes and joint space narrowing (KL 3-4) within one year (Driban et al., 2020). Therefore, the recent guidelines on KOA emphasize the prevention and treatment of KOA in the early stages of knee pain and joint function problems (Roos and Arden, 2016). As it becomes important to prevent and manage discomfort, such as knee pain from osteoarthritis, herbal medicines or functional foods with anti-inflammatory properties have begun to be developed as safe alternative choices. *Tamarindus Indica* seeds (Rao et al., 2019), Curcuma longa L. (Calderón-Pérez et al., 2021), Krill oil (Suzuki et al., 2016) and Rosehip (Ginnerup-Nielsen et al., 2015), which have antioxidant and anti-inflammatory properties, have been reported to improve knee joint pain associated with KOA in the initial state.

PF, an intervention in our study, is one of the common foods in East Asia and has been traditionally used as a medicinal herb (Ahmed, 2018). PF is composed of approximately 400 different bioactive compounds, including perillaldehyde (Ahmed and Tavaszi-Sarosi, 2019), anthocyanins (He et al., 2015), terpenoids (Akihisa et al., 2006), and coumarins (Liu et al., 2017) etc., and has demonstrated strong anti-inflammatory (Urushima et al., 2015), antioxidant (Assefa et al., 2018), neuroprotective (Senavong et al., 2016), anticancer (Kwak and Ju, 2015), and hepatoprotective effects (Yang et al., 2013) due to these compounds. Previous studies have demonstrated that PF provided anti-arthritic effects by reducing the arthritis score and neutrophil-to-lymphocyte ratio in a Balb/c collagen-induced arthritis model based on its anti-inflammatory and antioxidant effects (Jin et al., 2019). In this current study, although PFE improved the knee joint pain and function, it did not show a significant effect on the five inflammatory markers (COMP, COX-2, neutrophil and lymphocyte ratio, HS-CRP, and ESR), which are secondary outcome measurements (Table 3). There was no significant change probably because the inflammatory markers of the participants in this study were in the normal range at baseline.

Although several related studies have been conducted *in vitro* and *in vivo*, there has been no clinical evaluation of PF for relieving knee joint pain yet. The current RCT confirmed that PFE significantly mitigated knee pain and improved knee joint function. This study has few limitations and complementary points for further study. First, to reduce the placebo effect and confirm the changes of inflammation-related markers in PFE, it is necessary to include participants with KOA assessed by K-L grade 1 or higher in the inclusion criteria of participants. Second, investigation and analysis of disease duration and amount of exercise affecting the recovery of knee joint pain should be performed. Despite these limitations, this study is meaningful enough in that it is the first clinical trial to scientifically present the efficacy of PFE on pain reduction and functional improvement of the knee joint.

## 5 Conclusion

The current findings suggest that taking PFE for eight weeks is more effective than placebo on reducing knee joint pain assessed by VAS scores and improving knee joint function assessed by WOMAC scores in sub-healthy people with mild knee joint pain. There were no significant differences between PFE and placebo on inflammatory laboratory examinations. PFE accompanied no major concerns on safety assessment compared to placebo.

## Data Availability

The original contributions presented in the study are included in the article/Supplementary Material, further inquiries can be directed to the corresponding authors.

## References

[B1] Ahmed H. (2018). Ethnomedicinal, phytochemical and pharmacological investigations of Perilla frutescens (L.) britt. Molecules 24 (1), 102. 10.3390/molecules24010102 30597896 PMC6337106

[B2] Ahmed H. M. Mohan Al-Zubaidy A. Othman-Qadir G. (2022). Biological investigations on macro-morphological characteristics, polyphenolic acids, antioxidant activity of Perilla frutescens (L) Britt. grown under open field. Saudi J. Biol. Sci. 29 (5), 3213–3222. 10.1016/j.sjbs.2022.01.059 35844372 PMC9280211

[B3] Ahmed H. M. Tavaszi-Sarosi S. (2019). Identification and quantification of essential oil content and composition, total polyphenols and antioxidant capacity of Perilla frutescens (L.) Britt. Food Chem. 275, 730–738. 10.1016/j.foodchem.2018.09.155 30724256

[B4] Akihisa T. Kamo S. Uchiyama T. Akazawa H. Banno N. Taguchi Y. (2006). Cytotoxic activity of Perilla frutescens var. japonica leaf extract is due to high concentrations of oleanolic and ursolic acids. J. Nat. Med. 60 (4), 331–333. 10.1007/s11418-006-0015-9

[B5] Altman R. Asch E. Bloch D. Bole G. Borenstein D. Brandt K. (1986). Development of criteria for the classification and reporting of osteoarthritis. Classification of osteoarthritis of the knee. Diagnostic and Therapeutic Criteria Committee of the American Rheumatism Association. Arthritis rheumatism 29 (8), 1039–1049. online. 10.1002/art.1780290816 3741515

[B6] Ameye L. G. Chee W. S. (2006). Osteoarthritis and nutrition. From nutraceuticals to functional foods: A systematic review of the scientific evidence. Arthritis Res. Ther. 8 (4), R127. [online]. 10.1186/ar2016 16859534 PMC1779427

[B7] Assefa A. D. Jeong Y.-J. Kim D.-J. Jeon Y.-A. Ok H.-C. Baek H.-J. (2018). Characterization, identification, and quantification of phenolic compounds using UPLC-Q-TOF-MS and evaluation of antioxidant activity of 73 Perilla frutescens accessions. Food Res. Int. 111, 153–167. 10.1016/j.foodres.2018.05.017 30007672

[B8] Bellamy N. Wilson C. Hendrikz J. (2009). Population-based normative values for the Western Ontario and McMaster (WOMAC®) osteoarthritis index and the Australian/Canadian (AUSCAN) hand osteoarthritis index functional subscales. Inflammopharmacology 18 (1), 1–8. 10.1007/s10787-009-0021-0 20024627

[B9] Blagojevic M. Jinks C. Jeffery A. Jordan K. P. (2010). Risk factors for onset of osteoarthritis of the knee in older adults: A systematic review and meta-analysis. Osteoarthr. Cartil. 18 (1), 24–33. [online]. 10.1016/j.joca.2009.08.010 19751691

[B10] Calderón-Pérez L. Llauradó E. Companys J. Pla-Pagà L. Boqué N. Puiggrós F. (2021). Acute effects of turmeric extracts on knee joint pain: A pilot, randomized controlled trial. J. Med. Food 24 (4), 436–440. 10.1089/jmf.2020.0074 32749918 PMC8080919

[B11] Chiarotto A. Maxwell L. J. Ostelo R. W. Boers M. Tugwell P. Terwee C. B. (2019). Measurement properties of visual analogue scale, numeric rating scale, and pain severity subscale of the brief pain inventory in patients with low back pain: A systematic review. J. Pain 20 (3), 245–263. 10.1016/j.jpain.2018.07.009 30099210

[B12] Conaghan P. G. Dickson J. Grant R. L. Guideline Development Group (2008). Care and management of osteoarthritis in adults: Summary of NICE guidance. BMJ 336 (7642), 502–503. 10.1136/bmj.39490.608009.ad 18310005 PMC2258394

[B13] Cui A. Li H. Wang D. Zhong J. Chen Y. Lu H. (2020). Global, regional prevalence, incidence and risk factors of knee osteoarthritis in population-based studies. EClinicalMedicine 29-30, 100587. 10.1016/j.eclinm.2020.100587 34505846 PMC7704420

[B14] Darlow B. Brown M. Thompson B. Hudson B. Grainger R. McKinlay E. (2018). Living with osteoarthritis is a balancing act: An exploration of patients’ beliefs about knee pain. BMC Rheumatol. 2 (1), 15. 10.1186/s41927-018-0023-x 30886966 PMC6390552

[B15] Driban J. B. Eaton C. B. Lo G. H. Price L. L. Lu B. Barbe M. F. (2015). Overweight older adults, particularly after an injury, are at high risk for accelerated knee osteoarthritis: Data from the osteoarthritis initiative. Clin. Rheumatol. 35 (4), 1071–1076. 10.1007/s10067-015-3152-2 26686368 PMC4811718

[B16] Driban J. B. Harkey M. S. Barbe M. F. Ward R. J. MacKay J. W. Davis J. E. (2020). Risk factors and the natural history of accelerated knee osteoarthritis: A narrative review. BMC Musculoskelet. Disord., 21, 332, [online]. 21. 10.1186/s12891-020-03367-2 32471412 PMC7260785

[B17] Dye S. F. (2003). Functional morphologic features of the human knee: An evolutionary perspective. Clin. Orthop. Relat. Res. 410, 19–24. 10.1097/01.blo.0000063563.90853.23 12771813

[B18] Enck P. Klosterhalfen S. (2019). Placebos and the placebo effect in drug trials. Concepts Princ. Pharmacol. 260, 399–431. 10.1007/164_2019_269 31463606

[B19] Faul F. Erdfelder E. Buchner A. Lang A.-G. (2009). Statistical power analyses using G*Power 3.1: Tests for correlation and regression analyses. Behav. Res. Methods 41 (4), 1149–1160. 10.3758/brm.41.4.1149 19897823

[B20] Felson D. T. (2013). Osteoarthritis as a disease of mechanics. Osteoarthr. Cartil. 21 (1), 10–15. 10.1016/j.joca.2012.09.012 PMC3538894 23041436

[B21] Ginnerup-Nielsen E. Christensen R. Bliddal H. Zangger G. Hansen L. Henriksen M. (2015). Improved gait in persons with knee related mobility limitations by a rosehip food supplement: A randomized, double-blind, placebo-controlled trial. Gait Posture 42 (3), 340–347. 10.1016/j.gaitpost.2015.07.001 26234471

[B22] Guo Y. Yang P. Liu L. (2018). Origin and efficacy of hyaluronan injections in knee osteoarthritis: Randomized, double-blind trial. Med. Sci. Monit. 24, 4728–4737. 10.12659/msm.908797 29983409 PMC6069440

[B23] Hartmann H. Wirth K. Klusemann M. (2013). Analysis of the load on the knee joint and vertebral column with changes in squatting depth and weight load. Sports Med. 43 (10), 993–1008. [online]. 10.1007/s40279-013-0073-6 23821469

[B24] He Y.-K. Yao Y.-Y. Chang Y.-N. (2015). Characterization of anthocyanins in Perilla frutescens var. acuta extract by advanced UPLC-ESI-IT-TOF-MSn method and their anticancer bioactivity. Molecules 20 (5), 9155–9169. 10.3390/molecules20059155 25996217 PMC6272396

[B25] Jang S. Lee K. Ju J. H. (2021). Recent updates of diagnosis, pathophysiology, and treatment on osteoarthritis of the knee. Int. J. Mol. Sci. 22 (5), 2619. 10.3390/ijms22052619 33807695 PMC7961389

[B26] Jin C. H. So Y. Kim H.-Y. Han S. N. Kim J.-B. (2019). Anti-arthritic activities of supercritical carbon dioxide extract derived from radiation mutant Perilla frutescens var. Crispa in collagen antibody-induced arthritis. Nutrients 11 (12), 2959. 10.3390/nu11122959 31817175 PMC6950222

[B27] Jin C. So Y. Nam B. Han S. Kim J.-B. (2017). Isoegomaketone alleviates the development of collagen antibody-induced arthritis in male balb/c mice. Molecules 22 (7), 1209. 10.3390/molecules22071209 28753954 PMC6152219

[B28] Kim H. M. Nam B. Paudel S. B. Nam J.-W. Han A.-R. Jeong H. G. (2020). 9-Hydroxy-isoegomaketone inhibits LPS-induced NO and inflammatory cytokine production in RAW264.7 cells. Mol. Med. Rep. 23 (3), 181. 10.3892/mmr.2020.11820 PMC7809899 33398364

[B29] Kim I. J. Kim H. A. Seo Y.-I. Jung Y. O. Song Y. W. Jeong J. Y. (2011). Prevalence of knee pain and its influence on quality of life and physical function in the Korean elderly population: A community based cross-sectional study. J. Korean Med. Sci. 26 (9), 1140–1146. 10.3346/jkms.2011.26.9.1140 21935267 PMC3172649

[B30] Kim Y.-R. Nam B. Han A.-R. Kim J.-B. Jin C. H. (2021). Isoegomaketone from Perilla frutescens (L.) britt stimulates MAPK/ERK pathway in human keratinocyte to promote skin wound healing. Evidence-Based Complementary Altern. Med. 2021, 6642606–6642608. 10.1155/2021/6642606 PMC7889401 33628306

[B31] Kim Y. Kim A.-Y. Jo A. Choi H. Cho S.-S. Choi C. (2017). Development of user-friendly method to distinguish subspecies of the Korean medicinal herb Perilla frutescens using multiplex-PCR. Molecules 22 (4), 665. 10.3390/molecules22040665 28430157 PMC6154563

[B32] Kirsch I. (2013). The placebo effect revisited: Lessons learned to date. Complementary Ther. Med. 21 (2), 102–104. 10.1016/j.ctim.2012.12.003 23497811

[B33] Knee, Definition of KNEE. [online] Available at: www.nice.org.uk [Accessed 22 Nov. 2022]. (2014).

[B34] Kwak Y. Ju J. (2015). Inhibitory activities of Perilla frutescensbritton leaf extract against the growth, migration, and adhesion of human cancer cells. Nutr. Res. Pract. 9 (1), 11–16. 10.4162/nrp.2015.9.1.11 25671062 PMC4317473

[B35] Kwon K. H. Kim K. I. Jun W. J. Shin D. H. Cho H. Y. Hong B. S. (2002). *In vitro* and *in vivo* effects of macrophage-stimulatory polysaccharide from leaves of Perilla frutescens var. crispa. Biol. Pharm. Bull. 25 (3), 367–371. 10.1248/bpb.25.367 11913535

[B36] Lajoie Y. Gallagher S. P. (2004). Predicting falls within the elderly community: Comparison of postural sway, reaction time, the berg balance scale and the activities-specific balance confidence (ABC) scale for comparing fallers and non-fallers. Archives Gerontology Geriatrics 38 (1), 11–26. 10.1016/s0167-4943(03)00082-7 14599700

[B37] Lane N. E. Brandt K. Hawker G. Peeva E. Schreyer E. Tsuji W. (2011). OARSI-FDA initiative: Defining the disease state of osteoarthritis. Osteoarthr. Cartil. 19 (5), 478–482. 10.1016/j.joca.2010.09.013 21396464

[B38] Lee J. W. Kang S. H. Choi H. G. (2021). Analysis of the associations between arthritis and fall histories in Korean adults. Int. J. Environ. Res. Public Health 18 (7), 3758. 10.3390/ijerph18073758 33916869 PMC8038444

[B39] Liu Y. Liu X.-H. Zhou S. Gao H. Li G.-L. Guo W.-J. (2017). Perillanolides A and B, new monoterpene glycosides from the leaves of Perilla frutescens. Rev. Bras. Farmacogn. 27 (5), 564–568. 10.1016/j.bjp.2017.06.003

[B40] Makino T. Nakamura T. Ono T. Muso E. Honda G. (2001). Suppressive effects of Perilla frutescens on mesangioproliferative glomerulonephritis in rats. Biol. Pharm. Bull. 24 (2), 172–175. 10.1248/bpb.24.172 11217087

[B41] Meng L. Lozano Y. Gaydou E. Li B. (2008). Antioxidant activities of polyphenols extracted from Perilla frutescens varieties. Molecules 14 (1), 133–140. 10.3390/molecules14010133 19127243 PMC6253943

[B42] Morozzi G. Fabbroni M. Bellisai F. Cucini S. Simpatico A. Galeazzi M. (2007). Low serum level of COMP, a cartilage turnover marker, predicts rapid and high ACR70 response to adalimumab therapy in rheumatoid arthritis. Clin. Rheumatol. 26 (8), 1335–1338. 10.1007/s10067-006-0520-y 17285224

[B43] Netter F. H. Dalley A. F. (2003). Atlas of human anatomy. Teterboro, N. J: Icon Learning Systems.

[B44] Nguyen U.-S. D. T. Zhang Y. Zhu Y. Niu J. Zhang B. Felson D. T. (2011). Increasing prevalence of knee pain and symptomatic knee osteoarthritis: Survey and cohort data. Ann. Intern. Med. 155 (11), 725–732. [online]. 10.7326/0003-4819-155-11-201112060-00004 22147711 PMC3408027

[B45] Nice Clinical Guidelines, 2022, Osteoarthritis: Care and management | guidance | NICE. [online] Available at: https://www.nice.org.uk/guidance/cg177/chapter/Recommendations#%20diagnosis-2 [Accessed 22 Nov. 2*022* ].

[B46] Peat G. McCaRney R. Croft P. (2001). Knee pain and osteoarthritis in older adults: A review of community burden and current use of primary health care. Ann. Rheumatic Dis. 60 (2), 91–97. 10.1136/ard.60.2.91 PMC1753462 11156538

[B47] Previtali D. Capone G. Marchettini P. Candrian C. Zaffagnini S. Filardo G. (2022). High prevalence of pain sensitization in knee osteoarthritis: A meta-analysis with meta-regression. CARTILAGE 13 (1), 19476035221087698. 10.1177/19476035221087698 35356833 PMC9137298

[B48] Rao P. S. Ramanjaneyulu Y. S. Prisk V. R. Schurgers L. J. (2019). A combination of *Tamarindus indica* seeds and Curcuma longa rhizome extracts improves knee joint function and alleviates pain in non-arthritic adults following physical activity. Int. J. Med. Sci. 16 (6), 845–853. 10.7150/ijms.32505 31337958 PMC6643110

[B49] Roos E. M. Arden N. K. (2016). Strategies for the prevention of knee osteoarthritis. Nat. Rev. Rheumatol. 12 (2), 92–101. [online]. 10.1038/nrrheum.2015.135 26439406

[B50] Salaffi F. Leardini G. Canesi B. Mannoni A. Fioravanti A. Caporali R. (2003). Reliability and validity of the western Ontario and McMaster Universities (WOMAC) osteoarthritis index in Italian patients with osteoarthritis of the knee. Osteoarthr. Cartil. 11 (8), 551–560. 10.1016/s1063-4584(03)00089-x 12880577

[B51] Senavong P. Kongkham S. Saelim S. Suangkavathin V. (2016). Neuroprotective effect of Perilla extracts on PC12 cells. Planta Medica 81 (S 01), S1–S381. 10.1055/s-0036-1596545 29927179

[B52] Suzuki Y. Fukushima M. Sakuraba K. Sawaki K. Sekigawa K. (2016). Krill oil improves mild knee joint pain: A randomized control trial. PloS One 11 (10), e0162769. [online]. 10.1371/journal.pone.0162769 27701428 PMC5049853

[B53] Svensson P. Miles T. S. Graven-Nielsen T. Arendt-Nielsen L. (2000). Modulation of stretch-evoked reflexes in single motor units in human masseter muscle by experimental pain. Exp. Brain Res. 132 (1), 65–71. 10.1007/s002210000335 10836636

[B54] Takahashi M. Sugiyama Y. Kawabata K. Takahashi Y. Irie K. Murakami A. (2011). 1,2-Di-*O*-α-linolenoyl-3-*O*-β-galactosyl-*sn*-glycerol as a superoxide generation inhibitor from*Perilla frutescens*var.*crispa* . Biosci. Biotechnol. Biochem. 75 (11), 2240–2242. 10.1271/bbb.110414 22056448

[B55] Urushima H. Nishimura J. Mizushima T. Hayashi N. Maeda K. Ito T. (2015). *Perilla frutescens* extract ameliorates DSS-induced colitis by suppressing proinflammatory cytokines and inducing anti-inflammatory cytokines. Am. J. Physiology-Gastrointestinal Liver Physiology 308 (1), G32–G41. 10.1152/ajpgi.00294.2014 25359539

[B56] Wang J. (2017). Efficacy and safety of adalimumab by intra-articular injection for moderate to severe knee osteoarthritis: An open-label randomized controlled trial. J. Int. Med. Res. 46 (1), 326–334. 10.1177/0300060517723182 28840750 PMC6011328

[B57] World Health Organization (2002). The asia-pacific perspective: Redefining obesity and its treatment. Regional Office For The Western Pacific, International Association For The Study Of Obesity and International Obesity Task Force, Available at: www.merriam-webster.com .

[B58] Yang S.-Y. Hong C.-O. Lee G. P. Kim C.-T. Lee K.-W. (2013). The hepatoprotection of caffeic acid and rosmarinic acid, major compounds of Perilla frutescens, against t-BHP-induced oxidative liver damage. Food Chem. Toxicol. 55, 92–99. 10.1016/j.fct.2012.12.042 23306788

[B59] Zdzieblik D. Oesser S. Gollhofer A. Koenig D. (2017). Corrigendum: Improvement of activity-related knee joint discomfort following supplementation of specific collagen peptides. Appl. Physiology, Nutr. Metabolism 42 (11), 1237. 10.1139/apnm-2017-0693 29081241

[B60] Zeni J. A. Axe M. J. Snyder-Mackler L. (2010). Clinical predictors of elective total joint replacement in persons with end-stage knee osteoarthritis. BMC Musculoskelet. Disord. 11 (1), 86. 10.1186/1471-2474-11-86 20459622 PMC2877653

