# Supplementary material for: Efficacy of Perilla frutescens (L.) Britton var. frutescens extract on mild knee joint pain: A randomized controlled trial
Source: Front Pharmacol. 2023 Mar 14;14:1114410. doi: 10.3389/fphar.2023.1114410 (PMC10043449; doi:10.3389/fphar.2023.1114410)
Supplement: Supplementary file 1 [file Table1.DOCX]

| **Supplementary Table 1. Adverse events reported in each group (safety set)** | | | |
| --- | --- | --- | --- |
| Classification of adverse events | Adverse events | PFE (n=40) | Placebo (n=40) |
| Oral problem (2 cases) | Tongue discomfort | 1 | 0 |
|  | Wisdom tooth extraction | 0 | 1 |
| Digestive system problem (3 cases) | Gastritis | 1* | 1 |
|  | Hemorrhoids | 0 | 1 |
| Respiratory system problem (3 cases) | Common cold | 0 | 1 |
|  | Stuffy nose | 1 | 0 |
|  | Enteritis | 0 | 1 |
| Head problem (3 cases) | Headache | 2 | 0 |
|  | Migraine | 1* | 0 |
| Skin problem (1 case) | Eczema | 1 | 0 |
| Gynecological problem (1 case) | Menstrual pain | 0 | 1 |
| Musculoskeletal problem (1 case) | Wrist discomfort | 0 | 1 |
| Circulatory system problem (1 case) | High blood pressure | 0 | 1 |
| Total number of participants who had adverse event (15 cases) | | 6 | 8 |
| * One participant complained of symptoms of headache and gastritis and 6 participants reported adverse event in the PFE group. | | | |
