# Supplementary material for: Efficacy of Perilla frutescens (L.) Britton var. frutescens extract on mild knee joint pain: A randomized controlled trial
Source: Front Pharmacol. 2023 Mar 14;14:1114410. doi: 10.3389/fphar.2023.1114410 (PMC10043449; doi:10.3389/fphar.2023.1114410)
Supplement: Supplementary file 2 [file Table2.DOCX]

| **Supplementary Table 2. Changes in primary outcome according to subgroups within the PFE group** | | | | | |  |  |
| --- | --- | --- | --- | --- | --- | --- | --- |
| Sub-group | | VAS | WOMAC | | | | |
|  |  |  | total | pain | stiffness | function | |
| Male (N=12) | | 18.8 ± 12.5 | 14.6 ± 9.8 | 2.9 ± 2.2 | 1.3 ± 1.2 | 10.3 ± 7.9 | |
| Female (N=24) | | 20.0 ± 10.3 | 23.5 ± 16.0 | 5.1 ± 3.3 | 2.2 ± 1.6 | 16.2 ± 12.2 | |
| Difference | | -1.1 ± 11.4 | -8.9 ± 13.3^*^ | -2.2 ± 2.8^*^ | -0.9 ± 1.4 | -5.9 ± 10.3 | |
| Age > 45 (N=17) | | 17.2 ± 9.3 | 17.0 ± 11.9 | 3.8 ± 2.9 | 1.6 ± 1.4 | 11.5 ± 8.9 | |
| Age ≤ 45 (N=19) | | 21.7 ± 12.0 | 23.7 ± 16.6 | 3.4 ± 4.9 | 2.2 ± 1.6 | 16.6 ± 12.6 | |
| Difference | | -4.4 ± 10.7 | -6.7 ± 8.6 | -1.1 ± 2.3 | -0.5 ± 6.4 | -5.1 ± 6.3 | |
| BMI > 23 (N=21) | | 19.2 ± 11.6 | 15.9 ± 9.7 | 3.4 ± 2.2 | 1.4 ± 1.4 | 11.0 ± 7.5 | |
| BMI ≤ 23 (N=15) | | 20.1 ± 10.2 | 27.1 ± 18.2 | 5.8 ± 3.8 | 2.6 ± 1.5 | 18.7 ± 14.0 | |
| Difference | | -0.9 ± 10.9 | -11.2 ± 14.6^*^ | -2.4 ± 3.1^*^ | -1.2 ± 1.4^*^ | -7.6 ± 11.2^*^ | |
| Data presented as mean ± standard deviation.  If *p*-value > 0.05 at Shapiro-Wilk test, differences are analyzed between groups by an independent two-sample t-test.  If *p*-value < 0.05 at Shapiro-Wilk test, differences are analyzed between groups by Mann-Whitney test.  * *p* < 0.05, **: *p* < 0.01, ***: *p* < 0.001 indicates statistical significance between subgroups.  VAS: Visual Analogue Scale, WOMAC: Western Ontario and McMaster Universities Osteoarthritis score. | | | | | | | |
