# Supplementary figures and images for: Efficacy of Perilla frutescens (L.) Britton var. frutescens extract on mild knee joint pain: A randomized controlled trial
Source: Front Pharmacol. 2023 Mar 14;14:1114410. doi: 10.3389/fphar.2023.1114410 (PMC10043449; doi:10.3389/fphar.2023.1114410)

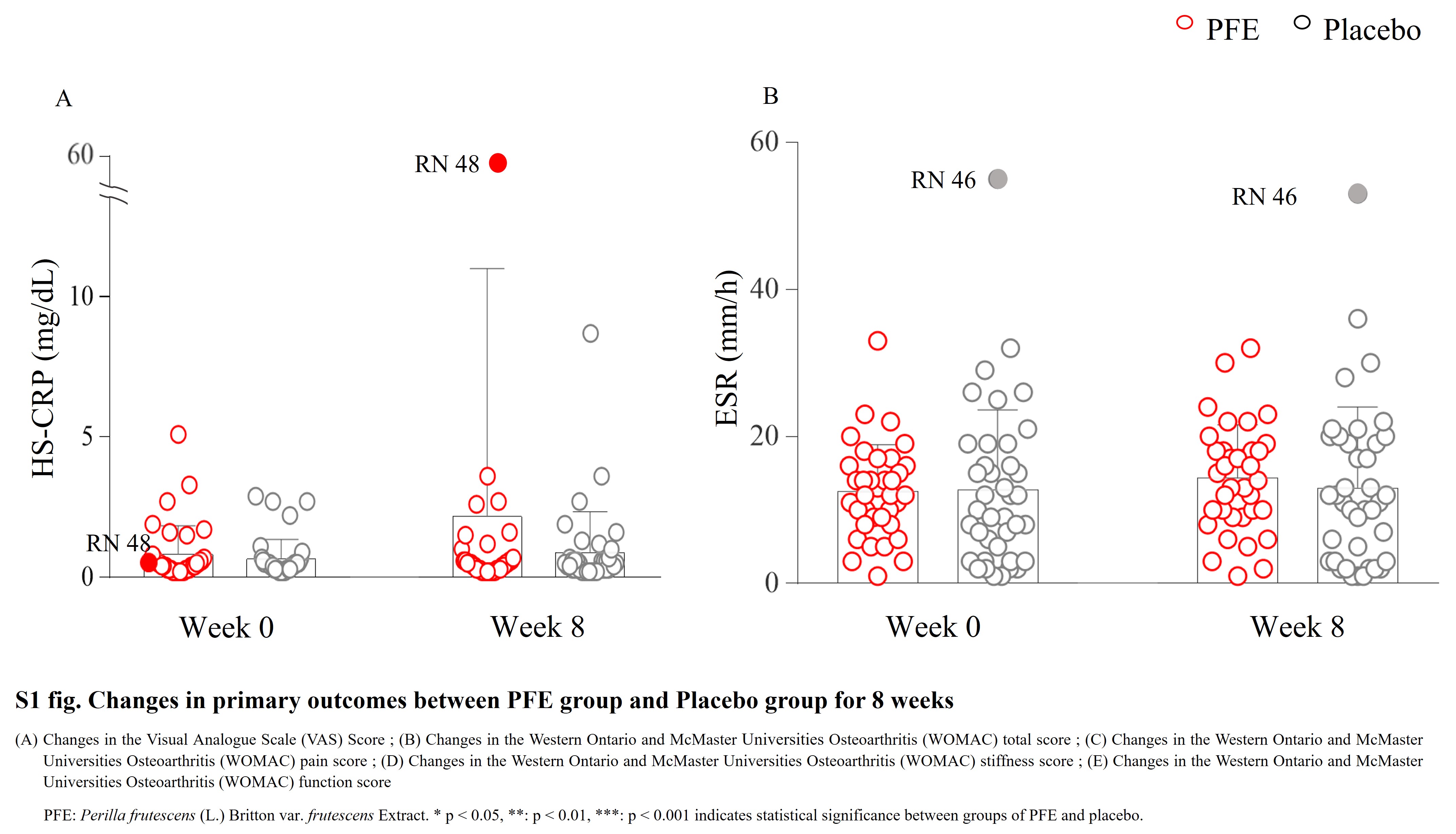

Supplement: Supplementary file 3 [file Image1.jpg]
